# Supplementary material for: Genetic Factors Causing Thyroid Dyshormonogenesis as the Major Etiologies for Primary Congenital Hypothyroidism: Clinical and Genetic Characterization of 33 Patients
Source: J Clin Med. 2022 Dec 9;11(24):7313. doi: 10.3390/jcm11247313 (PMC9786654; doi:10.3390/jcm11247313)
Supplement: Supplementary file 1 [file jcm-11-07313-s001.zip › Supplementary document/Supplementary Table 1.docx]

**Supplementary Table 1.** Genetic findings of the 11 CH patients with unclear etiology

| Patient  No. | CH-related  Genes | Genotypes |
| --- | --- | --- |
| 23 | *DUOX2* | c.2654G>A (p.Arg885Gln)/－ |
| 24 | － | －/－ |
| 25 | *TG* | c.7822G>A (p.Val2608Ile)/－ |
| 26 | － | －/－ |
| 27 | *TG*; *DUOX2* | [c.2149C>T(p.Arg717*); c.4027C>T(p.Leu1343Phe)]/－ |
| 28 | － | －/－ |
| 29 | － | －/－ |
| 30 | － | －/－ |
| 31 | － | －/－ |
| 32 | － | －/－ |
| 33 | － | －/－ |

－: no variants were found.
