# Supplementary material for: Genetic Factors Causing Thyroid Dyshormonogenesis as the Major Etiologies for Primary Congenital Hypothyroidism: Clinical and Genetic Characterization of 33 Patients
Source: J Clin Med. 2022 Dec 9;11(24):7313. doi: 10.3390/jcm11247313 (PMC9786654; doi:10.3390/jcm11247313)
Supplement: Supplementary file 1 [file jcm-11-07313-s001.zip › Supplementary document/Supplementary Table 2.docx]

**Supplementary Table 2.** Pathogenicity prediction of the three novel missense variants

| In silico tools | Scores (pathogenicity prediction) for the novel missense variants | | |
| --- | --- | --- | --- |
|  | c.3904T>C in *DUOX2* | c.7404G>C in *TG* | c.307T>A in *PAX8* |
| PolyPhen-2 | 1.000 (probably damaging) | 0.995 (probably damaging) | 0.278 (benign) |
| Mutation Taster | 0.999 (disease-causing) | 0.999 (disease-causing) | 0.999 (disease-causing) |
| PROVEN | -13.36 (deleterious) | -2.5 (deleterious) | -5.611 (deleterious) |
| SIFT | 0.000 (deleterious) | 0.000 (deleterious) | 0.04 (deleterious) |
| MutationAssessor | 3.735 (high) | 0.583 (medium) | 0.111 (neutral) |
