# Supplementary material for: Genetic Factors Causing Thyroid Dyshormonogenesis as the Major Etiologies for Primary Congenital Hypothyroidism: Clinical and Genetic Characterization of 33 Patients
Source: J Clin Med. 2022 Dec 9;11(24):7313. doi: 10.3390/jcm11247313 (PMC9786654; doi:10.3390/jcm11247313)
Supplement: Supplementary file 1 [file jcm-11-07313-s001.zip › Supplementary document/Supplementary Table 3.docx]

**Supplementary Table 3.** Comparative alignment of the primate peptides homologous for human *DUOX2*

| **Primates** | **Peptides** | **From** | **Amino acid sequences** | **To** |
| --- | --- | --- | --- | --- |
| Human | ENST00000389039 | 1296 | YKSGQWVRIACLALGTTEYHP | 1317 |
| Olive baboon | ENSPANP00000013999 | 1299 | YKSGQWVRIACLALGTTEYHP | 1320 |
| Tarsier | ENSTSYP00000012966 | 1296 | YKSGQWVRIACLALGTNEYHP | 1317 |
| Coquerel's sifaka | ENSPCOP00000014556 | 1301 | YKSGQWVRIACLALGTTEYHP | 1322 |
| Bushbaby | ENSOGAP00000011441 | 1296 | YKSGQWVRIACLALGTTEYHP | 1317 |
| Orangutan | ENSPPYP00000007295 | 1296 | YKSGQWVRIACLALGTTEYHP | 1317 |
| Bolivian squirrel monkey | ENSSBOP00000006750 | 1296 | YKSGQWVRIACLALGTTEYHP | 1317 |
| Gibbon | ENSNLEP00000007015 | 1296 | YKSGQWVRIACLALGTTEYHP | 1317 |
| Sooty mangabey | ENSCATP00000033032 | 1296 | YKSGQWVRIACLALGTTEYHP | 1317 |
| Chimpanzee | ENSPTRP00000012012 | 1296 | YKSGQWVRIACLALGTTEYHP | 1317 |
| Macaque | ENSMMUP00000011460 | 1296 | YKSGQWVRIACLALGTTEYHP | 1317 |
| Mouse Lemur | ENSMICP00000008364 | 1307 | YKSGQWVRIACLALGTTEYHP | 1328 |
| Gorilla | ENSGGOP00000011422 | 1296 | YKSGQWVRIACLALGTTEYHP | 1317 |
| Greater bamboo lemur | ENSPSMP00000028713 | 1302 | YKSGQWVRIACLALGTTEYHP | 1324 |
| Crab-eating macaque | ENSMFAP00000013999 | 1331 | YKSGQWVRIACLALGTTEYHP | 1352 |
| Ma's night monkey | ENSANAP00000010212 | 1296 | YKSGQWVRIACLALGTTEYHP | 1317 |
| Pig-tailed macaque | ENSMNEP00000025111 | 1296 | YKSGQWVRIACLALGTTEYHP | 1317 |
| Golden snub-nosed monkey | ENSRROP00000043324 | 1296 | YKSGQWVRIACLALGTTEYHP | 1317 |
| Black snub-nosed monkey | ENSRBIP00000001909 | 1296 | YKSGQWVRIACLALGTTEYHP | 1317 |
| Marmoset | ENSCJAP00000004206 | 1296 | YKSGQWVRIACLALGTTEYHP | 1317 |
| Capuchin | ENSCCAP00000033600 | 1296 | YKSGQWVRIACLALGTTEYHP | 1317 |
| Vervet-AGM | ENSCSAP00000005906 | 1282 | YKSGQWVRIACLALGTTEYHP | 1303 |
| Bonobo | ENSPPAP00000038501 | 1003 | YKSGQWVQIACLALGTTEYHP | 1024 |
| Drill | ENSMLEP00000027586 | 981 | YKSGQWVQIACLALGTTEYHP | 1002 |

The conserved tryptophan (W) is in red. All species of amino acid is conserved.
