# Supplementary material for: Genetic Factors Causing Thyroid Dyshormonogenesis as the Major Etiologies for Primary Congenital Hypothyroidism: Clinical and Genetic Characterization of 33 Patients
Source: J Clin Med. 2022 Dec 9;11(24):7313. doi: 10.3390/jcm11247313 (PMC9786654; doi:10.3390/jcm11247313)
Supplement: Supplementary file 1 [file jcm-11-07313-s001.zip › Supplementary document/Supplementary Table 4.docx]

**Supplementary Table 4.** Comparative alignment of the primate peptides homologous for human *PAX8*

| **Primates** | **Peptides** | **From** | **Amino acid sequences** | **To** |
| --- | --- | --- | --- | --- |
| Human | ENSP00000395498 | 96 | RQNPTMFAWEIRDRLLAEGVC | 117 |
| Coquerel's sifaka | ENSPCOP00000016480 | 96 | RQNPTMFAWEIRDRLLAEGVC | 117 |
| Crab-eating macaque | ENSMFAP00000045638 | 96 | RQNPTMFAWEIRDRLLAEGVC | 117 |
| Olive baboon | ENSPANP00000003667 | 96 | RQNPTMFAWEIRDRLLAEGVC | 117 |
| Gelada | ENSTGEP00000035798 | 96 | RQNPTMFAWEIRDRLLAEGVC | 117 |
| Chimpanzee | ENSPTRP00000085551 | 96 | RQNPTMFAWEIRDRLLAEGVC | 117 |
| Black snub-nosed monkey | ENSRBIP00000019786 | 96 | RQNPTMFAWEIRDRLLAEGVC | 117 |
| Capuchin | ENSCCAP00000040121 | 96 | RQNPTMFAWEIRDRLLAEGVC | 117 |
| Angola colobus | ENSCANP00000032631 | 96 | RQNPTMFAWEIRDRLLAEGVC | 117 |
| Bolivian squirrel monkey | ENSSBOP00000008184 | 96 | RQNPTMFAWEIRDRLLAEGVC | 117 |
| Tarsier | ENSTSYP00000011507 | 36 | RQNPTMFAWEIRDRLLAEGVC | 57 |
| Bonobo | ENSPPAP00000016463 | 96 | RQNPTMFAWEIRDRLLAEGVC | 117 |
| Golden snub-nosed monkey | ENSRROP00000025886 | 96 | RQNPTMFAWEIRDRLLAEGVC | 117 |
| Ma's night monkey | ENSANAP00000008837 | 96 | RQNPTMFAWEIRDRLLAEGVC | 117 |
| Gibbon | ENSNLEP00000048797 | 96 | RQNPTMFAWEIRDRLLAEGVC | 117 |
| Sumatran orangutan | ENSPPYP00000040531 | 96 | RQNPTMFAWEIRDRLLAEGVC | 117 |
| Mouse Lemur | ENSMICP00000037379 | 96 | RQNPTMFAWEIRDRLLAEGVC | 117 |
| Sooty mangabey | ENSCATP00000026422 | 96 | RQNPTMFAWEIRDRLLAEGVC | 117 |
| Pig-tailed macaque | ENSMNEP00000026753 | 96 | RQNPTMFAWEIRDRLLAEGVC | 117 |
| Macaque | ENSMMUP00000015207 | 96 | RQNPTMFAWEIRDRLLAEGVC | 117 |
| White-tufted-ear marmoset | ENSCJAP00000066815 | 96 | RQNPTMFAWEIRDRLLAEGVC | 117 |
| Vervet-AGM | ENSCSAP00000012737 | 96 | RQNPTMFAWEIRDRLLAEGVC | 117 |

The conserved Phenylalanine (F) is in red. All species of amino acid is conserved.
