# Supplementary material for: Genetic Factors Causing Thyroid Dyshormonogenesis as the Major Etiologies for Primary Congenital Hypothyroidism: Clinical and Genetic Characterization of 33 Patients
Source: J Clin Med. 2022 Dec 9;11(24):7313. doi: 10.3390/jcm11247313 (PMC9786654; doi:10.3390/jcm11247313)
Supplement: Supplementary file 1 [file jcm-11-07313-s001.zip › Supplementary document/Supplementary Table 5.docx]

**Supplementary Table 5.** Comparative alignment of the primate peptides homologous for human *TG*

| **Primates** | **Peptides** | **From** | **Amino acid sequences** | **To** |
| --- | --- | --- | --- | --- |
| Human | ENSP00000220616 | 2461 | VLNDAQTKLLAVSGPFHYWGP | 2481 |
| Marmoset | ENSCJAP00000008448 | 2463 | VLNDAQTKLLAVSGPFHYWSP | 2483 |
| Bolivian squirrel monkey | ENSSBOP00000019418 | 2463 | VLNDAQTKLLAVSGPFHYWSP | 2483 |
| Pig-tailed macaque | ENSMNEP00000010586 | 2462 | ILNDAQTKLLAVSGPFHYWGP | 2482 |
| Tarsier | ENSTSYP00000000182 | 2463 | ALNDAQTKLLAVSGPFHYWGP | 2483 |
| Olive baboon | ENSPANP00000032197 | 2462 | ILNDAQTKLLAVSGPFHYWGP | 2482 |
| Ma's night monkey | ENSANAP00000038964 | 2463 | VLNDAQTKLLAVSGPFHYWSP | 2483 |
| Capuchin | ENSCCAP00000022064 | 2463 | VLNDAQTKLLAVSGPFHYWSP | 2483 |
| Gibbon | ENSNLEP00000007024 | 2468 | VLNDAQTKLLAVSGPFHYWGP | 2488 |
| Golden snub-nosed monkey | ENSRROP00000029754 | 2462 | ILNDAQTKLLAVSGPFHYWGP | 2482 |
| Sooty mangabey | ENSCATP00000034680 | 2462 | ILNDAQTKLLAVSGPFHYWGP | 2482 |
| Greater bamboo lemur | ENSPSMP00000006303 | 2463 | ILNDAQTKLLAVSGPFHYWGP | 2483 |
| Gorilla | ENSGGOP00000005495 | 2462 | VLNDAQTKLLAVSGPFHYWGP | 2482 |
| Mouse Lemur | ENSMICP00000000949 | 2209 | VLNDAQTKLLAVSGPFHYWGP | 2229 |
| Drill | ENSMLEP00000033850 | 2462 | ILNDAQTKLLAVSGPFHYWGP | 2482 |
| Black snub-nosed monkey | ENSRBIP00000022074 | 1467 | ILNDAQTKLLAVSGPFHYWGP | 1487 |
| Macaque | ENSMMUP00000046539 | 2320 | ILNDAQTKLLAVSGPFHYWGP | 2340 |
| Bushbaby | ENSOGAP00000008741 | 2465 | ILNDAQTKLLAVSGPFHYWGP | 2485 |
| Orangutan | ENSPPYP00000021197 | 1594 | VLNDAQTKLLAVSGPFHYWGP | 1614 |
| Bonobo | ENSPPAP00000021502 | 2462 | VLNDAQTKLLAVSGPFHYWGP | 2482 |
| Chimpanzee | ENSPTRP00000035225 | 2462 | VLNDAQTKLLAVSGPFHYWGP | 2482 |
| Vervet-AGM | ENSCSAP00000006114 | 2480 | ILNDAQTKLLAVSGPFHYWGP | 2500 |

The conserved lysine (K) is in red. All species of amino acid is conserved.
