# Supplementary material for: Genetic Factors Causing Thyroid Dyshormonogenesis as the Major Etiologies for Primary Congenital Hypothyroidism: Clinical and Genetic Characterization of 33 Patients
Source: J Clin Med. 2022 Dec 9;11(24):7313. doi: 10.3390/jcm11247313 (PMC9786654; doi:10.3390/jcm11247313)
Supplement: Supplementary file 1 [file jcm-11-07313-s001.zip › Supplementary document/Supplementary Table 6.docx]

**Supplementary Table 6.** DQ evaluation in 33 CH patients on clinic follow up

| Patients | Actual age(m) | Evaluation contents | | | | Developmental quotient (DQ) | Test scale |
| --- | --- | --- | --- | --- | --- | --- | --- |
|  |  | Personal- social skill | Fine  moter | Language | Gross motor |  |  |
| 1 | 20 | 98 | 76 | 76 | 106 | 86 | DDST |
| 2 | 6 | 90 | 98 | 115 | 98 | 100 | DDST |
| 3 | NA | NA | NA | NA | NA | NA | DDST |
| 4 | NA | NA | NA | NA | NA | NA | DDST |
| 5 | 87 | 87 | 93 | 96 | 95 | 97 | GMDS |
| 6 | 30 | 90 | 100 | 90 | 110 | 96 | DDST |
| 7 | 25 | 89 | 83 | 71 | 89 | 86 | DDST |
| 8 | 9 | 111 | 100 | 89 | 83 | 96 | DDST |
| 9 | 14 | 110 | 99 | 96 | 88 | 99 | DDST |
| 10 | 18 | 81 | 97 | 89 | 97 | 96 | DDST |
| 11 | 53 | 99 | 88 | 88 | 109 | 95 | DDST |
| 12 | 75 | 96 | 96 | 96 | 104 | 98 | DDST |
| 13 | 58 | 84 | 94 | 99 | 105 | 93 | DDST |
| 14 | 36 | 101 | 101 | 107 | 95 | 101 | DDST |
| 15 | 36 | 92 | 84 | 100 | 92 | 93 | DDST |
| 16 | 53 | 97 | 102 | 97 | 102 | 99 | DDST |
| 17 | 48 | 99 | 84 | 87 | 99 | 93 | DDST |
| 18 | 42 | 91 | 91 | 91 | 98 | 94 | DDST |
| 19 | 9 | 99 | 99 | 85 | 99 | 98 | DDST |
| 20 | 14 | 101 | 83 | 83 | 83 | 87 | DDST |
| 21 | 54 | 67 | 59 | 50 | 89 | 64 | DDST |
| 22 | 26 | 86 | 98 | 98 | 98 | 95 | DDST |
| 23 | 10 | 74 | 95 | 95 | 74 | 86 | DDST |
| 24 | 24 | 88 | 88 | 82 | 100 | 87 | DDST |
| 25 | － | － | － | － | － | － | － |
| 26 | － | － | － | － | － | － | － |
| 27 | 73 | 99 | 99 | 95 | 99 | 98 | DDST |
| 28 | 36 | 100 | 95 | 100 | 100 | 99 | DDST |
| 29 | 4 | 111 | 89 | 111 | 89 | 96 | DDST |
| 30 | 33 | 92 | 102 | 102 | 102 | 100 | DDST |
| 31 | 31 | 100 | 100 | 100 | 100 | 100 | DDST |
| 32 | 19 | 87 | 95 | 95 | 95 | 90 | DDST |
| 33 | 13 | 100 | 82 | 94 | 105 | 98 | DDST |

NA: Data not accessible; －: Not performed; GMDS: Griffiths Mental Development Scales; DDST: Denver Developmental Screening Tests; The scales analysis indicates normal development if the DQ>85.
