# Supplementary figures and images for: Genetic Factors Causing Thyroid Dyshormonogenesis as the Major Etiologies for Primary Congenital Hypothyroidism: Clinical and Genetic Characterization of 33 Patients
Source: J Clin Med. 2022 Dec 9;11(24):7313. doi: 10.3390/jcm11247313 (PMC9786654; doi:10.3390/jcm11247313)

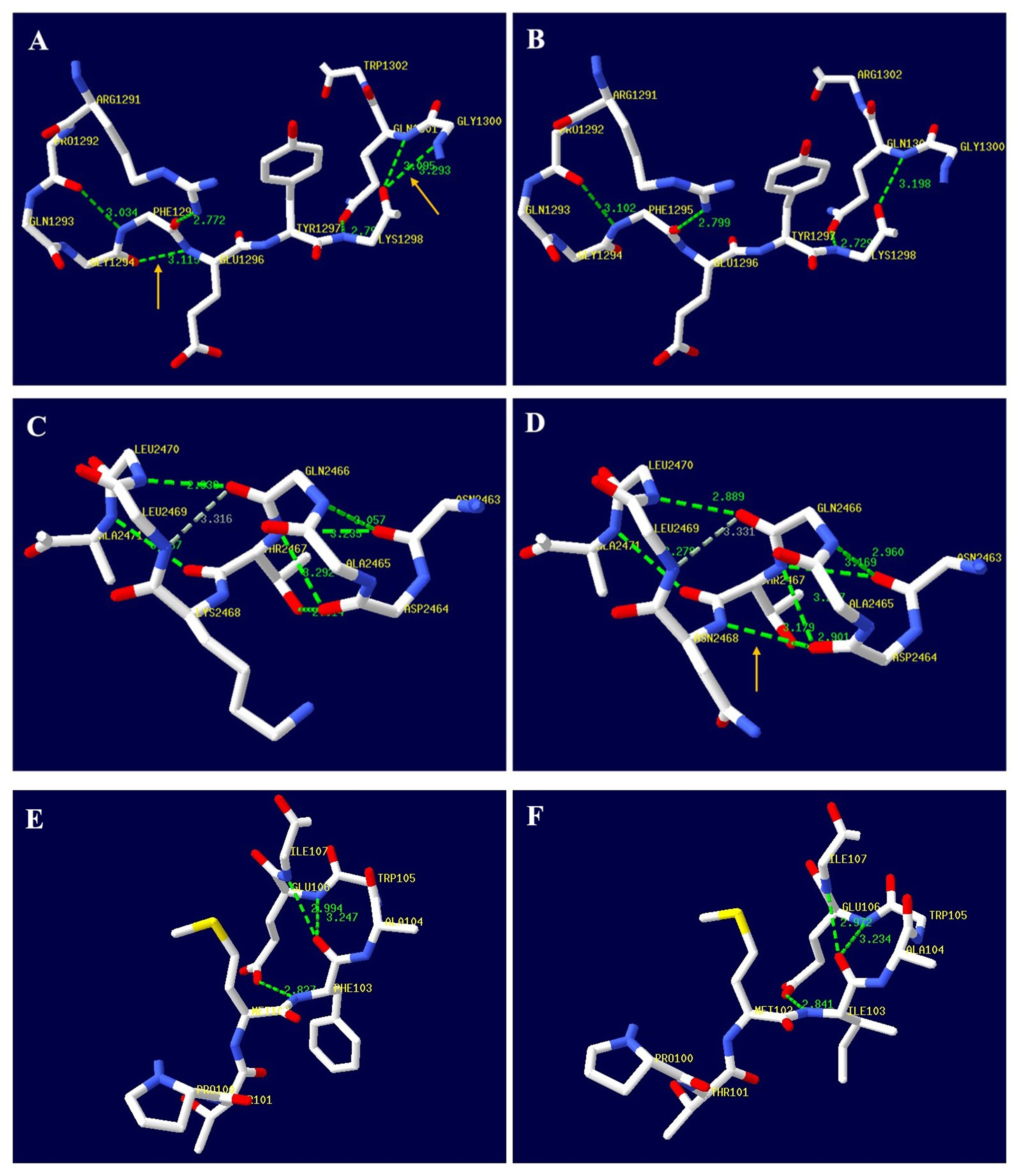

Supplement: Supplementary file 1 [file jcm-11-07313-s001.zip › Supplementary document/Supplementary Figure 1.jpg]
